# Moving beyond referrals to strengthen late-life depression care: a qualitative examination of primary care clinic and community-based organization partnerships

**DOI:** 10.1186/s12913-022-07997-1

**Published:** 2022-05-06

**Authors:** Jenny Wagner, Stuart Henderson, Theresa J. Hoeft, Melissa Gosdin, Ladson Hinton

**Affiliations:** 1 Evaluation Specialist, School of Medicine Office of Research, University of California, Davis, 2921 Stockton Blvd. Suite 1400, Sacramento, CA 95817 USA; 2 Director, Evaluation, School of Medicine Office of Research, University of California, Davis, 2921 Stockton Blvd. Suite 1400, Sacramento, CA 95817 USA; 3 Research Assistant Professor, Department of Psychiatry and Behavioral Sciences, University of Washington, 1959 NE Pacific Street, Seattle, WA 98195-6560 USA; 4 Qualitative Research Analyst, Center for Healthcare Policy and Research, University of California, Davis, 2103 Stockton Blvd., Suite 2224, Sacramento, CA 95817 USA; 5 Department of Psychiatry and Behavioral Sciences, University of California, Davis, 2230 Stockton Blvd, Sacramento, CA 95817 USA

**Keywords:** Depression, Older adults, Collaborative care, Social determinants of health, Multi-sector collaborations, Qualitative research

## Abstract

**Background:**

National guidelines have called for greater integration of primary care and behavioral health services, with more recent attention to social care and community-based services. Under growing resource constraints healthcare organizations have tended to rely on referrals to external entities to address social care needs. Traditional referral models, however, may not be equipped to provide for the complex needs of older adults with depression. The Care Partners Project was designed to strengthen late-life depression care through integrated partnerships between primary care clinics and community-based organizations. We sought to understand how these integrated partnerships, with shared tasks and accountability across organizations, changed the nature of depression care for older adults.

**Methods:**

We conducted 65 in-depth, semi-structured interviews and six focus groups with service providers involved in the project, including care managers, primary care providers, and psychiatric consultants, and applied inductive and deductive qualitative thematic analysis to develop themes around participants’ experiences with the partnered initiative.

**Results:**

We found the partnerships established by the Care Partners Project reshaped late-life depression care in two ways: (1) bidirectional communication across organizations facilitated greater recognition among providers of intersecting medical and social needs associated with late-life depression; and (2) depression care became more coordinated and effective as care teams established or strengthened relationships across organizations.

**Conclusions:**

These findings highlight the ways cross-organizational health and social care partnerships that move beyond traditional referrals can strengthen late-life depression care and enhance organizational capacities.

## Background

Depression is a leading cause of disability in high-income countries, affecting roughly 15% of older adults in the United States [1–3]. Older adults are particularly vulnerable to depression due to increased loneliness, limited social support, unmet basic resource needs, and comorbid physical health conditions which may limit normal functionality [4, 5]. However, depression is frequently misdiagnosed and undertreated within this population, likely related to normalization of the disease as part of the aging process [6].

Identifying and treating depression in late life are particularly complex when social challenges are present [7]. A range of factors within economic, social and physical environments shape mental health over the life course and act as either barriers or facilitators to access and utilization of depression care [8]. For example, many older adults with depression face economic, food and housing insecurities, as well as limited access to healthcare services, reliable transportation, health insurance, and social support [9–11]. These social factors are often addressed separately from medical care given limited capacities within healthcare systems, as well as perceptions among physicians of a lack of control over the social conditions affecting their patients’ lives [12].

About half of patients with depression will solely be seen in primary care settings as opposed to specialized mental or behavioral health systems [13]. Addressing these mental health concerns in tandem with related unmet social needs presents several challenges for primary care providers (PCPs). For example, PCPs may be unsure of their role in addressing social needs or have difficulty in distinguishing between depression and “normal” distress stemming from social issues [7]. PCPs have reported challenges in balancing their role in addressing patients’ medical and social needs, particularly given constraints of time, expertise, and resources [14]. They may also lack confidence in their ability to sufficiently treat depression or connect patients to supportive services in the community [7, 15, 16].

Several strategies have surfaced in recent years to support PCPs in providing depression care to patients [17]. Integrating primary care and behavioral health services, such as by establishing interprofessional teams within the healthcare system, has been shown to enhance depression care and improve outcomes [15, 18]. For example, the evidence-based collaborative care model builds capacity for depression treatment in primary care settings through multidisciplinary teams of PCPs with support from care managers and psychiatric consultants. This approach has been shown to improve depression outcomes over usual care in over 90 randomized controlled trials and several meta-analyses [19–21]. However, even integrated primary and behavioral healthcare models are often unable to address many of the social and basic resource needs that both put patients at greater risk for depression and prevent them from accessing supportive services [9].

As healthcare providers alone cannot tackle the diversity of needs stemming from complex social and structural conditions, recent national guidelines recommend broader integration of healthcare, social care, and community-based services [22, 23]. However, many existing initiatives operate under a referral model, in which healthcare providers refer patients out to external community-based services [18]. While community-based organizations (CBOs) often have the expertise and resource networks to address a broad range of social needs, referral systems may not provide the level of navigation and follow-up needed to prevent patients from falling through the cracks. Studies suggest high-need patients are better able to utilize these community-based services with more intensive navigation support and coordination between service providers [24, 25].

Under growing attention to integrated health and social care in recent years, the Care Partners Project was initiated in 2015 by the Archstone Foundation to build on collaborative care and strengthen depression care for adults 65 years and older through primary care partnerships with CBOs. The Care Partners Project incorporated community-based social services in depression care beyond a traditional referral model by sharing tasks and accountability across organizations, as well as by extending outreach and services into community settings where older adults with depression may be more comfortable engaging in treatment. Although research has shown that integrated care may lead to better utilization of services, few studies explore how partnered care beyond traditional referral models may affect clinic and CBO providers’ and their organizations’ capacity to address depression care. In this article, we examine how the clinic-CBO partnerships formalized by the Care Partners Project impacted late-life depression care processes and coordination between health and social care providers.

## Methods

### Data collection

The multi-site Care Partners Project engaged two cohorts of clinic-CBO partnerships, which we refer to as “sites,” between 2015–2021 and represented locations throughout California. As part of an evaluation of the Care Partners Project, we conducted annual in-depth interviews and focus groups with selected PCPs, care managers, administrators, and psychiatric consultants from the Care Partners sites. A purposive sampling strategy was used to ensure key actors (i.e., those most actively engaged in the Care Partners Project) from each clinic and CBO were represented in the evaluation. The Care Partners evaluation team based at the University of California, Davis conducted semi-structured interviews and focus groups at multiple time points over the course of the project. The interview and focus group discussion guides were developed for the Care Partners Project and are provided in the supplemental materials. These guides were adapted over the course of the project depending on whether a participant had been interviewed previously. Interviewers (authors 1, 2, 4, 5) included graduate-level trained (MPH, PhD, MD) male and female health researchers. Our analysis draws on 65 interviews representing 43 unique informants from clinics and CBOs across both cohorts, conducted between April 2019 and April 2021, as well as six focus groups conducted in September 2020 (four groups) and May 2021 (two groups). Eleven participants were interviewed twice during this time period. Participant demographics are as follows: 60% female, 35% male, 5% decline to state; 49% white, 23% Hispanic/Latino, 12% black or African American, and 7% Asian or Pacific Islander, and 9% other or decline to state. All participants had at least some college education, and 80% had at least a bachelor’s degree.

Focus group participants and interviewees were recruited through an email message and informed of the purpose of the study, the topics to be discussed, and who would be interviewing them. All participants were given the option to decline participation. None declined participation, although two were unreachable. Focus group attendees, many of whom had also participated in individual interviews, were assigned to separate groups based on role and organization type (i.e., clinic or CBO). All interviews and focus groups were conducted virtually through video conference or telephone by trained evaluators and lasted approximately 60 min. Only the evaluators and participants were present for the interviews. The researchers had no prior relationships with participants prior to the study. Interviews and focus groups were digitally recorded and transcribed for analysis. Interviewers took notes during the interviews and debriefed with the study team afterwards.

The Institutional Review Boards at the University of California, Davis and University of Washington determined the evaluation to be quality improvement and therefore exempt from human subjects review. Participants gave verbal consent for their participation in all interviews and focus groups and were free to decline participation at any time.

### Data analysis

We analyzed interview and focus group data using inductive and deductive qualitative thematic analysis [26, 27]. An initial set of index codes was developed a priori based on the research questions and key informant interview guide. Four members of the research team identified index codes which were then discussed as a group and refined. Each team member coded a selection of interview transcripts to norm the coding process and assess differences in application of the initial coding guide. Coding issues were discussed and resolved to reach interpretive congruence [28]. After the initial review of the transcripts, an inductive coding approach was applied which allowed patterns and intersections between codes to be identified and themes to be developed [29]. The research team wrote analytic memos and met weekly to discuss emerging themes until data saturation was reached. Finally, themes were validated through input from the team based at University of Washington, which provided technical assistance, coaching and support for the sites over the course of the project. All transcripts were coded using QRS International’s NVivo 12 qualitative data analysis software (released 2019).

## Results

Our findings suggest the primary care clinic and CBO partnerships established by the Care Partners Project reshaped and strengthened late-life depression care in two ways: (1) bidirectional interpersonal communication across organizations facilitated greater recognition among providers of intersecting medical and social needs associated with late-life depression; and (2) depression care became more coordinated and effective as care teams established or strengthened relationships across organizations. The following sections describe these themes in greater detail and offer examples from a subset of Care Partners sites.

### Building awareness of intersections in medical and social needs

Within the Care Partners Project, healthcare and social service providers brought different lenses and priorities to patient care, often with distinct perspectives on medical and social needs as factors involved in patients’ depression symptoms and treatment. Clinical care providers were often focused on behavioral interventions and medication management, while social care providers from community-based organizations tended to prioritize patients’ social needs, in alignment with their respective organizational missions. The partnerships, however, crystalized for both clinic and CBO administrators and direct service providers the intersecting layers of patients’ medical and social needs. For example, service providers from Care Partners sites described the challenges their patients faced such as housing instability or housing quality issues, food and economic insecurities, lack of reliable transportation, and social isolation. They suggested these social needs often intersected and had a direct influence on older patients’ depression symptoms, as a clinic administrator reflected:

“We found that with a lot of our seniors … that it wasn't, I don't want to say it's not depression, it is depression, but it was related to some material need. And as soon as we were able to take care of that need, a lot of the depression went down. So, it wasn't like through the miracle of counseling or medication... it was actually the social service needs that have I think provided a lot of relief at least during COVID for sure” (site 1).

Many of the clinic-based providers and administrators were aware of their patients’ social needs before their partnerships with CBOs were established; however, the partnerships deepened their understanding and appreciation for the complexity of the relationship between depression and social needs. Another clinic administrator emphasized “you have to peel away the social determinants to really evaluate folks for their underlying depression” (site 2).

The partnership between clinics and CBOs also highlighted the constraints medical problems and functional limitations placed on patients’ engagement in activities or treatments that might have otherwise improved social connections and quality of life. In these cases, comorbid health conditions both contributed to depression symptoms and limited patients’ engagement in depression treatment. A CBO care manager reflected on this layering of medical and non-medical needs in relation to a patient’s depression, which constrained their options for addressing a patient’s social needs:

“Sometimes a doctor will refer a person and be like – she’s really depressed. She used to knit. Just have her start knitting again, you know? But then when you get to someone’s house, in reality, they’re like, you know, in bed 12 hours a day and they have arthritis in their hands. And they’re incontinent. And, so, the carpet’s stained with urine and, you know, there’s just so many other levels of things that need to happen before they would be in a place where they would wanna sit down and knit, you know?... It’s not usually that straightforward. Especially with how complex most of these seniors are that are getting referred to our team” (site 2).

In some cases, Care Partners sites came into the project already aware patients’ intersecting medical and social needs. The partnership, however brought the complexities and subtleties of the intersection of needs to the forefront. Particularly among clinic staff, the partnered initiative increased awareness of underlying social needs their patients faced that may not have been uncovered during a brief primary care visit. In some cases, the partnerships brought new capacities to address needs the clinics had known existed but were beyond their reach to intervene. A CBO care manager described the shift in their view of patients’ medical and social needs and, in recognition of these distinct yet interrelated factors, the importance of taking a whole-person approach to depression care:

“Sometimes it seems as though the learning process is that these are much more complex [cases] than anyone of us thought that they would be. Increasing your viewpoint, from just social service-ly stabilizing somebody or medically stabilizing somebody to the increased view of stabilizing the whole person. It doesn't seem like it would be a really big difference, but it's a big difference” (site 1).

A primary care provider echoed this shift to a broader recognition of both medical and social factors of depression that arose from their partnership, saying “we cannot operate in a vacuum; we cannot operate in the confines of the clinic without knowing what's happening upstream and downstream. That has become very, very clear to me (site 2). An administrator at the same clinic described the CBO as “our virtual extension… by being on the team together, all of a sudden we really start to see the continuum [of patient needs]” (site 2). The consistency of communication and bidirectional flow of information between primary care clinic and CBO staff facilitated growth in their awareness of patients’ multifaceted needs and appreciation for the complexities of their patients’ lives, ultimately allowing them to care for patients more comprehensively.

### Strengthening care coordination and quality

Greater awareness of intersecting needs translated to stronger care coordination and perceived quality of care on both sides of the partnerships. The clinic-CBO partnerships provided space for deeper collaboration and relationship-building across organizations, even among those with formerly established relationships. For example, the partnerships allowed CBOs to connect their patients more efficiently to needed medical services through person-to-person interactions rather than through documents alone. One CBO care manager described this change as “back door” access to the clinic, emphasizing, “we can serve somebody more quickly and more completely by having that connection” (site 2). Another CBO care manager reiterated this point, saying “it tends to be a more efficient help for the clients… because we can address both issues [depression and social needs] at the same time” (site 1).

Clinic-based providers often relied on their CBO partner to better their understanding of patients’ home environments and risk factors, allowing them to act on issues that may otherwise not have surfaced in a clinical setting. For example, a clinic care manager described a patient for whom their CBO partner was able to glean critical insights from visiting the patient in their home:

“We talk about every single patient we have. And [the CBO] give[s] us updates. And sometimes those updates are crucial... I had one patient who is so, so invested in managing her diabetes and her congestive heart failure... I had no idea that she was getting fast food. She was having fast food delivered because she couldn't get to the grocery store. She never told me that and she's been on service with me for years... [the CBO] told me and they got her signed up for Meals on Wheels, and now Meals on Wheels brings two meals a day. It's diabetic meals and it's food she likes. So, I would never had known that if they hadn't been the eyes or ears there in the home to tell me that this was an issue… She probably didn't want to tell me that she's eating fast food three meals a day. And it never occurred for me to ask that” (site 3).

Likewise, clinic staff were often able to provide to their CBO partner context around patients’ physical or mental health, allowing them to better understand their patients’ limitations to engage in activities and treatment and subsequently to intervene in different ways, as described by a clinic administrator:

“We can also let [the CBO] know things that they may not know about this person related to their diagnoses or other things, that they may need to take into account why that person isn't following through because sometimes for community-based organizations, if somebody doesn't show up it's, "Oh, well, they're not following through. They're not compliant." Well, if we tell you that they're agoraphobic or they have mobility issues or other things that you may be unaware of, we'll go a different extra mile” (site 3).

Information sharing and coordination often occurred informally through email or impromptu conversations and formally in regular in-person or remote case review meetings, during which clinic and CBO staff discussed their different perspectives on each patient’s needs, as described by a clinic administrator:

“We already have a list of that [social needs] on our Excel spreadsheet, that we have internally, and so [the clinic care manager] is going to look to that, and go, "Okay [CBO care manager], was your team able to go out and provide the disability bar in [the patient’s] shower, or the smoke detectors," or whatever it is they need home repaired. Have you been able to go out and give her food, or check in with her? Then [the CBO] will turn around, and they have the same list, but from their perspective, and will tell us all the different social services and activities that they've had with her” (site 1).

These case review meetings, while often facilitated by clinic administrators or care managers, brought care providers from both clinic and CBO partners together to share their different perspectives and insights on patient needs and to coordinate services in ways unparalleled by most traditional referral models. In addition to facilitating information exchange and improving care coordination around patients’ medical and social needs, the clinic-CBO partnerships strengthened perceived quality of depression care they provided to patients. For example, a CBO administrator suggested the clinic care managers helped improve the quality of support CBO staff could offer to patients:

“The clinicians help the [CBO] to do their job better. And that if a [CBO care manager] was speaking about, "I'm frustrated I'm not making progress [with the patient]...” The clinicians could then speak to, "Well, let's talk a little bit about their diagnoses." And that helped understand their behavior... It helped the [CBO] to not be discouraged when they didn't feel they were making progress, but there also was a little bit of guidance in terms of their approach, if you will. And it was a beautiful back and forth” (site 4).

CBOs also enhanced the support clinics were able to provide to patients, for example, a clinic administrator described how their relationship with the CBO and better knowledge of the services they provided allowed them to connect patients more reliably with needed services:

“Now when we call and ask for a resource, there actually is one. Before, we used to make referrals, and often patients would go somewhere, and that item doesn't actually exist, or they've run out of it. Now, by building these personal relationships [with the CBO], it made a huge difference” (site 1).

## Discussion

In this article we highlight the ways in which primary care clinic and CBO partnerships changed the nature of late-life depression care for older adults. We found bridging perspectives and strengthening relationships across healthcare and community-based social service organizations allowed service providers on both sides of the partnerships to grow in awareness of the intersections in patients’ medical and social needs and to view patients through a whole person lens. The partnered approach and bidirectional sharing of information ultimately allowed providers from both primary care clinics and CBOs to gain a deeper understanding of their patients’ needs as well as their home and social environments. By involving a trusted CBO in depression care, some partnerships were able to break down barriers to care and reach patients that may otherwise have fallen through the cracks. For many cases described by interviewees, circumstances of everyday life impacted patients’ depression symptoms, their abilities to access treatment, and their responses to treatment. While referral models often involve unidirectional communication and limited follow-up between health and social care providers, the Care Partners Project introduced a more intensive back-and-forth sharing of information that strengthened relationships across organizations and enhanced care coordination and quality of care. These findings suggest transforming health and social care partnerships beyond referral models may strengthen depression care for older adults and have the potential to improve outcomes among the historically underserved populations often prioritized by community-based organizations.

Our findings are in alignment with previous studies which suggest social determinants of mental health and socially derived barriers to care must be mitigated in the delivery of an effective intervention for late-life depression [9]. For example, in their study of socioeconomic status and anxiety as predictors of response to depression treatment, Cohen and colleagues [2009] conclude “the social worlds which put older adults at elevated risk of depression also act to reduce the effectiveness of antidepressant treatments,” suggesting patients with fewer resources and greater levels of need may have more limited abilities to both access and benefit from depression care [30]. This conclusion fits within the broader context of Link and Phelan’s [1995] theory of fundamental causes, which links social conditions to health inequalities and calls for greater attention to the contextualization of risk factors in medical practice, [31] thereby “[avoiding] the enactment of interventions aimed at changing behaviors that are powerfully influenced by factors left untouched by the intervention” [32].

Involving social care organizations more intentionally in depression care workflows may support broader connections within diverse populations and enhance care quality and coordination particularly for patients with social needs. For example, Lahey, et al. describe the role of social workers in mitigating non-medical needs as a means to facilitate patients’ engagement in depression treatment [15]. These integrated care models which incorporate home-based social care are becoming particularly relevant as the U.S. population ages and more older adults develop comorbid, physically limiting medical conditions which both increase risk for depression and make access to treatment more difficult [33, 34].

Our study has several limitations. First, our findings draw only on interviews and focus groups with clinic and CBO staff and thus are not generalizable to other contexts or programs. Second, our reliance on qualitative data alone also precludes examination of depression care outcomes or direct comparison to other programs or models. Additionally, we do not incorporate patient perspectives in our analysis, which may have informed our understanding of changes in care quality or coordination as experienced by older adults enrolled in the Care Partners Project. We are also unable to examine the information provided to clinic and CBO staff from patients themselves regarding their care needs. Future research might illuminate patient perspectives on and experiences with integrated care teams involving multiple organizations. Finally, the Care Partners Project was possible because of funding provided by the Archstone Foundation, which established structures to promote regular collaboration and coordination between partnering organizations. Without such external funding and structure, organizations may need to find incentives to initiate and sustain their partnerships, particularly those with traditionally distinct missions and areas of service.

## Conclusions

Community-based organizations (CBOs) may be well-positioned to support primary care clinics in addressing the social factors of depression given their focus on social needs and often high-standing in lower-resourced communities [35, 36]. However, previous studies have suggested CBO and clinical partners are often misaligned in their understandings of capacity and demand, leaving CBOs to absorb high volumes of referrals they may not have capacity to address [37]. Thus, we argue cross-organizational partnerships that integrate social care services and establish lines of communication beyond traditional referral models may help to alleviate these deficits and improve care. Importantly, these cross-organizational partnerships are complex and often difficult to navigate considering the multiple organizational cultures and perspectives at play [38]. Primary care clinics and CBOs should take steps to ensure alignment of expectations and adequacy of funding streams to support all organizations’ work in the partnership prior to engaging in a partnered depression care initiative. Particularly considering the unique challenges of older adults, a movement toward community-based care models and social service partnerships may help to bolster capacities to serve the diverse and complex needs of an aging population.

## Data Availability

The qualitative data generated and analyzed during the current study are not publicly available to protect the identities and privacy of study participants. Data are however available from the authors upon reasonable request and with permission of Archstone Foundation.

## Abbreviations

CBO: Community-Based Organization
PCP: Primary Care Provider
